# Rapid calcium loss may cause arrhythmia in hemofiltration with regional citrate anticoagulation: a case report

**DOI:** 10.1186/s12882-018-0936-z

**Published:** 2018-06-14

**Authors:** Buyun Wu, Jing Wang, Guang Yang, Changying Xing, Huijuan Mao

**Affiliations:** Department of Nephrology, The First Affiliated Hospital of Nanjing Medical University, Nanjing, 210029 China

**Keywords:** Regional citrate anticoagulation, Hypercalcemia, Hypocalcemia, Arrhythmia, Threshold value

## Abstract

**Background:**

Renal replacement therapy (RRT) with regional citrate anticoagulation (RCA) is an important therapeutic approach for refractory hypercalcemia complicated with renal failure. However, RCA has the potential to induce arrhythmia caused by rapid calcium loss. We report a case of arrhythmia associated with rapid calcium loss during RCA-RRT.

**Case presentation:**

A 51-year-old man with hypercalcemia, primary hyperparathyroidism, and acute kidney injury was treated by predilutional-RCA-hemofiltration at a rate of 4.3 L/h. The effect of lowering serum calcium was unsatisfactory despite reducing calcium substitution gradually from 5.3 to 2.2 mmol/h in the first 8-h session of RCA-hemofiltration. New-onset sinus tachycardia with a prolonged QT interval occurred when calcium substitution was infused at rate of 1.1 mmol/h after 15 min of starting the second RCA-hemofiltration session (estimated net calcium loss was 7.54 mmol/h). When the calcium substitution was increased to usual rate of 5.6 mmol/h, the arrhythmia disappeared after 2 min. Arrhythmia did not recur when the calcium substitution rate was 2.2 mmol/h during the third session (estimated net calcium loss was 6.44 mmol/L). After the third RCA-hemofiltration session, the patient underwent parathyroidectomy and serum calcium returned to normal.

**Conclusions:**

This case indicated that rapid calcium loss may cause arrhythmia in RCA-hemofiltration, and the rate of net calcium loss should be limited below a threshold value to prevent similar adverse effect during RCA-RRT.

## Background

Regional citrate anticoagulation (RCA) is often recommended as a first-line anticoagulant for continuous renal replacement therapy (RRT) to reduce the risk of bleeding in critical ill patients and prolong the lifespan of hemofilters [1]. RRT with RCA is also an important therapeutic approach for refractory hypercalcemia [2]. However, rapid calcium loss in RCA has the potential to induce arrhythmia and hypotension. When the rate of calcium loss during RCA-RRT is beyond a threshold, arrhythmia may occur. Here, we describe a case of arrhythmia probably caused by rapid calcium loss in RCA-hemofiltration and address the hypothesis that a threshold rate of calcium loss could lead to arrhythmia.

## Case presentation

A 51-year-old man was admitted to the Department of Nephrology of the First Affiliated Hospital of Nanjing Medical University (Nanjing, China) in June 2016 because of hypercalcemia and renal failure. The patient was well until he developed persistent leg and low back pain 20 days before admission. Prior testing at a local hospital showed progressive abnormal renal function (serum creatinine, 304.2 μmol/L), a high serum calcium level (4.86 mmol/L), and an extremely high level of parathyroid hormone (PTH) (1551 ng/L; reference level, 12–88 ng/L). After hydration with saline and diuresis with loop diuretics and hemodialysis, the patient was transferred to our hospital. He had no unusual issues with the exception of a 1-year history of stage 3 chronic kidney disease.

**Fig. 1:**
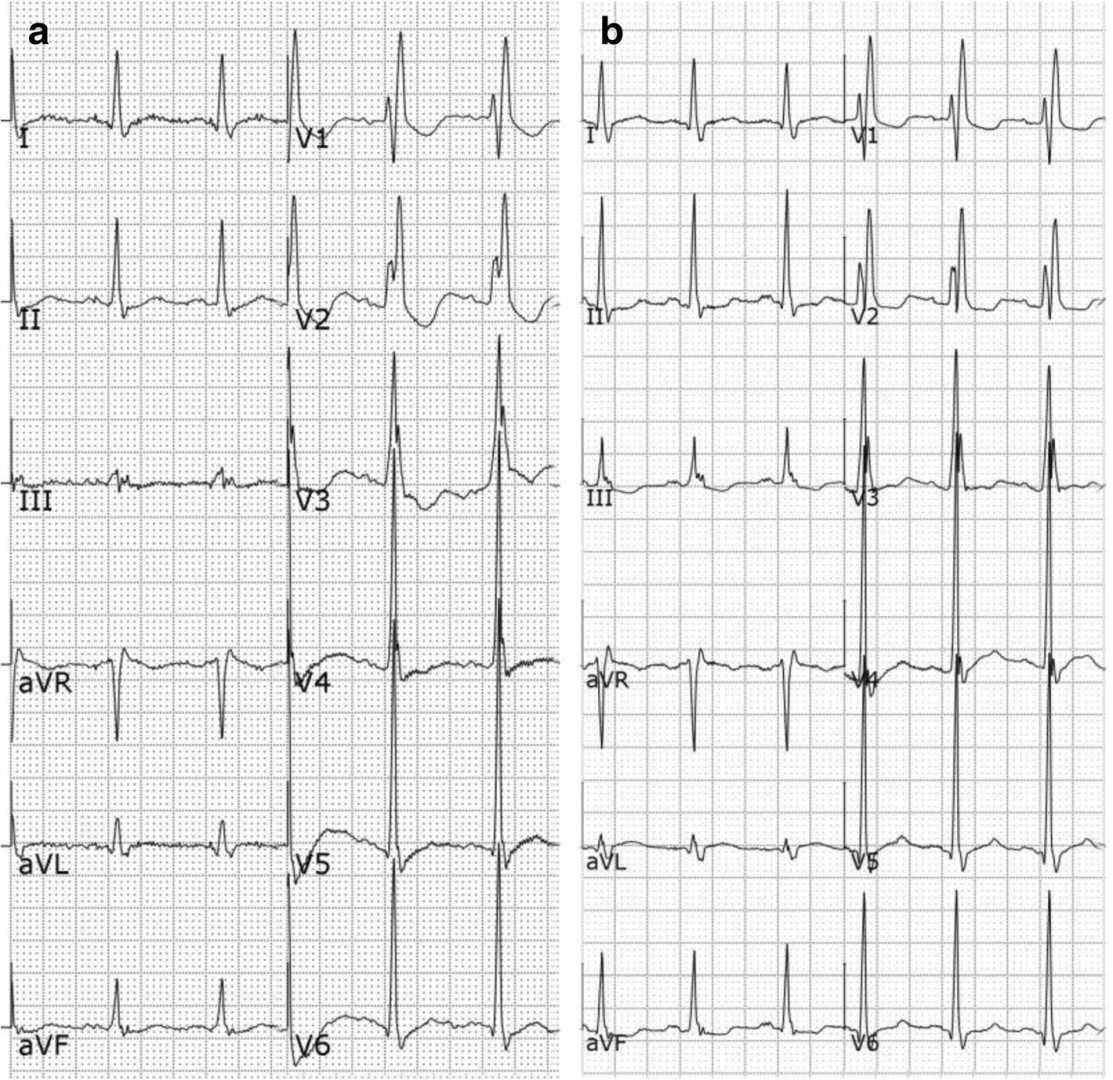
The QTc interval was prolonged during the second RCA-hemofiltration session, as compared to the first procedure. **a** Baseline ECG collected 3 days before. **b** ECG at the second RCA-hemofiltration session. The mean QT interval increased from 346 to 369 ms and the mean QTc interval increased from 423 to 494 ms

On admission, the patient reported fatigue, bone pain, and polyuria. A physical examination on admission revealed blood pressure of 119/80 mmHg and heart rate of 71 beats per min, but no specific findings of the lymph nodes, head, neck, heart, lung, abdomen, or joints. Laboratory testing revealed corrected serum calcium of 3.39 mmol/L, phosphorus of 0.84 mmol/L, PTH of 540 ng/mL, serum creatinine of 151 μmol/L (after hemodialysis), and hemoglobin of 120 g/L. Urine calcium was 9.2 mmol/24 h. An electrocardiogram (ECG) showed complete right bundle branch block and a precordial abnormal ST segment and normal corrected QT (QTc) interval (Fig. 1a). Myocardial marker analysis showed serum myoglobin of 31 μg/L and high-sensitivity troponin T of 826 ng/L. Ultrasonography revealed a nodule at the left lower pole of the parathyroid gland. Single-photon emission computed tomography results indicated an adenoma of the left lower pole of the parathyroid gland. Due to no family history of such disease and no medication history of thiazide or lithium, a diagnosis of primary hyperparathyroidism caused by a parathyroid adenoma was made. Because of the high level of troponin T and changes to the ST segment on an ECG, acute myocardium injury was considered, thus parathyroidectomy was deferred for multidisciplinary consultation. The final diagnoses were hypercalcemia, primary hyperparathyroidism, parathyroid adenoma, acute renal injury, chronic kidney disease, and acute myocardium injury.

**Fig. 2:**
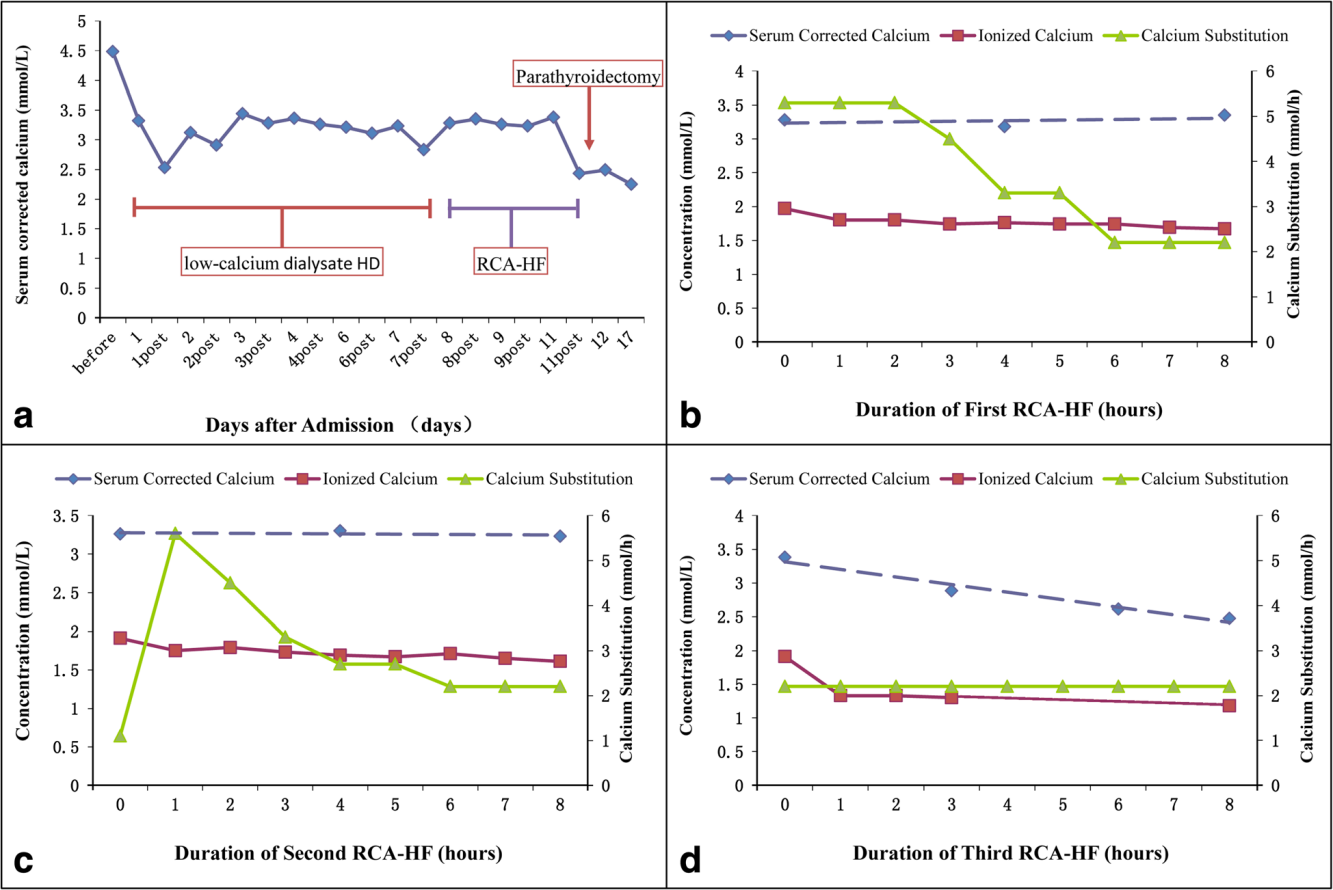
Changes in serum ionized calcium and calcium substitution rates during RCA-hemofiltration. **a** Changes in serum ionized calcium during hospitalization. **b-d** Changes in serum ionized calcium and calcium substitution rates during RCA-hemofiltration

Hydration with 3000 mL of fluid [60% saline (0.9%) and 40% dextrose (5%)] per day, furosemide at 40 mg three times per day, cinacalcet at 25 mg per day, and RRT were utilized to reduce serum calcium levels. Double lumen catheterization of the right femoral vein was used for vascular access. Slow extended dialysis with low-calcium dialysate (1.25 mmol) at bedside was started using a commercially available continuous RRT device (multiFiltrate, Fresenius Medical Care Deutschland GmbH, Bad Homburg, Germany). The dialysate flow was 4800 mL/h for 8 h each session. Changes in serum corrected calcium levels are shown in Fig. 2a. Due to an unsatisfactory lowering of serum calcium and preparation for parathyroidectomy, slow extended dialysis was switched to 8-h RCA-predilutional hemofiltration at a replacement rate of 4300 mL/h. The replacement solution was prepared by a local pharmacy (Na, 105 mmol/L; bicarbonate, 21.5 mmol/L; zero calcium; magnesium, 0.5 mmol/L; and glucose, 6.7 mmol/L). The infusion rate of 4% trisodium citrate (citrate dose 4.2 mmol/L) was 220 mL/h and the blood flow was 120 mL/min when hematocrit was 32%.

Given the time limit of treatment, we gradually reduced the rate of calcium supplementation from 5.6 to 2.2 mmol/h in the first session of RCA-hemofiltration (Fig. 2b). The post-filter ionized calcium was 0.34–0.53 mmol/L during RCA-hemofiltration. The corrected serum calcium level decreased from 3.5 to 3.2 mmol/L and ionized calcium decreased from 1.97 to 1.67 mmol/L. This result was not inspiring, despite estimated 30 mmol calcium loss during RCA-hemofiltration.

A lower infusion rate of calcium (1.1 mmol/h) was started at the second session of RCA-hemofiltration (Fig. 2c). However, chest discomfort and palpitations were reported by the patient after treatment for 15 min. At that time, his blood pressure was 110/64 mmHg and heart rate was 125 beats per min. Blood gas analysis showed potassium of 3.8 mmol/L and a rapid serum ionized calcium decrease (from 1.91 mmol/L to 1.71 mmol/L). Hence, calcium supplementation immediately increased to 5.6 mmol/h and 2 min later, the chest discomfort and palpitation disappeared. However, the 12-lead ECG demonstrated sinus tachycardia with a prolonged QTc interval as compared to baseline at 3 days before (Fig. 1 and Table 1). One hour later, the calcium infusion was gradually reduced to 2.2 mmol/h to reduce the level of serum calcium without inducing sinus tachycardia. The ionized calcium decreased from 1.91 to 1.61 mmol/L with unaltered corrected serum calcium.

**Table 1:** The QT and QTc intervals of two electrocardiograms (ECGs) of the patient

Parameters	ECG	I	II	III	aVR	aVL	aVF	V1	V2	V3	V4	V5	V6	Mean	Dispersion
QT intervals (ms)	a	348	339	346	342	342	335	335	356	356	351	353	346	346	21
	b	374	366	366	382	368	351	364	373	370	373	368	368	369	26
QTc interval (ms)	a	426	415	423	419	419	410	410	436	436	430	432	419	423	31
	b	502	491	491	512	493	471	488	500	496	500	493	493	494	41

The third RCA-hemofiltration session began at a calcium infusion rate of 2.2 mmol/h, which resulted in a decrease in corrected serum calcium from 3.5 to 2.7 mmol/L and in ionized calcium from 1.91 to 1.18 mmol/L without arrhythmia (Fig. 2d).

After the third RCA-hemofiltration session, high-sensitivity troponin T decreased to 89.8 ng/L and the patient was deemed sufficiently stable to undergo parathyroidectomy. An untypical parathyroid adenoma was pathologically confirmed. Postoperatively, PTH dropped to 16.9 ng/L and serum calcium dropped to 2.25 mmol/L. Serum calcium was 2.11 mmol/L and serum creatinine was 173 μmol/L at discharge. The patient received a telephone follow-up at 2 months after discharge and reported serum calcium of 2.13 mmol/L and serum creatinine of 112 μmol/L without calcium supplementation.

## Discussion

Hypercalcemia is a common medical problem with about 90% of cases caused by malignancy or primary hyperparathyroidism [3]. The mainstays of therapy for hypercalcemia include aggressive intravenous volume expansion with saline, bisphosphonate therapy, and administration of loop diuretics. Glucocorticoids or calcimimetics may also be considered, depending on the etiology of the hypercalcemia. For patients with renal failure and intolerant to aggressive intravenous volume expansion, RRT with calcium free or low calcium dialysate could be considered as an important therapy [4]. However, calcium-free hemodialysis is frequently associated with frequent cardiovascular adverse effects and is, therefore, seldom considered [5]. Successful treatment of hypercalcemia with hemofiltration and calcium-free replacement fluid has been described [6]. Several cases of successful treatment have also been reported using RRT with RCA and reduced rate of calcium supplementation [7–9]. Rebound hypercalcemia could occur following discontinuation of RRT with RCA. Therefore, treatment of the underlying cause of hypercalcemia is essential, while RRT is used as a temporizing measure to reduce extremely high levels of calcium.

Renal replacement therapy with RCA could help decrease serum calcium. Rapid calcium loss during renal replacement therapy may result in a rapid reduction of serum ionized calcium (from 1.91 to 1.71 mmol/L in 15 min in this patient). Low serum ionized calcium may affect repolarization, resulting in prolonged QT interval and triggering dysrhythmias [10]. In this case, a new-onset sinus tachycardia with prolonged QT interval occurred when the estimated rate of calcium loss was as high as 7.54 mmol/h. This was a typical ECG manifestation of hypocalcaemia despite a serum ionized calcium of 1.71 mmol/L. We did not consider the arrhythmia a result of transient cardiac ischemia because there were no obvious ST-segment changes on ECG, no blood pressure changes compared to that before hemofiltration, and the levels of high-sensitivity troponin T were considerably lower than that on admission (106 ng/L compared to 826 ng/L). Furthermore, there were no abnormalities in the levels of serum potassium or magnesium to explain the prolonged QT. We believe that the arrhythmia was associated with rapid calcium loss and rapid changes in serum ionized calcium during RCA-hemofiltration.

While correction of severe hypercalcemia is desired, rapid decrease in serum calcium may be associated with hypotension or arrhythmia. Our case with 3 sessions of RCA-hemofiltration showed a new-onset sinus tachycardia with prolonged QT interval occurred when the estimated loss rate of serum calcium was as high as 7.54 mmol/h, and arrhythmia did not occur when estimated calcium loss was 6.44 mmol/h (0.10 mmol/Kg/h). In previously published reports [6–9], in which estimated calcium losses did not exceed this threshold (Table 2), there were no reported events of arrhythmia. Conversely, a rapid and marked decline in calcium level, induced by a calcium-free dialysate, is associated with frequent adverse cardiovascular events [5]. Therefore, we suggest that the rate of calcium loss should not exceed 0.10 mmol/Kg/h.

**Table 2:** Recent studies of hypercalcemia treatment by prolonged intermittent or continuous RRT

Author, year	No.	Citrate dose^a^ (mmol/L)	Calcium dose^b^ (mmol/h)	Mode, blood flow (mL/min)	Dose (L/h)	Ionized Calcium (mmol/L)	Calcium loss^c^ (mmol/h)	Adverse effect
Low-calcium HD	Suppose ionized calcium reduced from 1.9 to 1.3 mmol/L after the dialyzer, blood flow was 150 mL/min and dialysate flow was 500 mL/min					1.90	5.40	No
Calcium-free HD [5]	Suppose ionized calcium reduced from 1.9 to 0.5 mmol/L after the dialyzer, blood flow was 150 mL/min and dialysate flow was 500 mL/min					1.90	12.6	Frequent
V.sramek 1998 [9]	1	3.7–6.1	0	CVVHDF, 100	3	2.0^d^	6.30	No
Mlles 2008 [8]	1	4.3	1.2	CVVHD, 200	2.2	1.77	2.91	No
Au 2012 [6]	1	–	0	CVVH, 150	2	2.19	4.38	No
Matis 2015 [7]	4	3	1.5–2.3	CVVHDF, 180	3.7	1.72	5.23	No
		3	1.3	CVVHDF, 100	2.5	1.90	3.70	Hypocalcemia
		3–3.5	2–2.75	CVVHDF, 100	2.5	1.79	2.48	No
		3	1.5	CVVHDF, 100	2.5	1.80	3.00	Hypocalcemia
		3	1.4	CVVHDF, 100	2.5	2.09	3.82	No
The present study	1							
	Second session	4.2	1.1(at start)	HF, 120	4.3	1.91	7.54	Arrhythmia
	Third session	4.2	2.2	HF, 120	4.3	1.91	6.44	No

Note: ^a^citrate (mmol/L) = infusion of citrate/blood flow; ^b^calcium dose (mmol/h) = infusion of calcium substitution; ^c^estimated calcium loss (mmol/h) = (ionized calcium + complex calcium) multiply by effluent – calcium dose, and suppose complex calcium = 0.1 mmol/L; ^d^supposed ionized calcium = 2.0 mmol/L

## Conclusion

In conclusion, this case report suggests rapid calcium removal may be associated with adverse events, such as arrhythmia. Our proposed threshold for calcium removal during RCA-RRT should be validated in clinical studies.

## Abbreviations

ECG: Electrocardiogram
PTH: Parathyroid hormonal
RCA: Regional citrate anticoagulation
RRT: Renal replacement therapy
